# Prognostic Stratification of Multiple Myeloma Using Clinicogenomic Models: Validation and Performance Analysis of the IAC-50 Model

**DOI:** 10.1097/HS9.0000000000000760

**Published:** 2022-08-02

**Authors:** Adrián Mosquera Orgueira, Marta Sonia González Pérez, José Ángel Díaz Arias, Beatriz Antelo Rodríguez, María-Victoria Mateos

**Affiliations:** 1 Health Research Institute of Santiago de Compostela (IDIS), Santiago de Compostela, Spain; 2 Complexo Hospitalario Universitario de Santiago de Compostela (CHUS), Department of Hematology, SERGAS, Santiago de Compostela, Spain; 3 Hospital Universitario de Salamanca, Instituto de Investigación Biomédica de Salamanca (IBSAL), Centro de Investigación del Cancer (IBMCC-USAL, CSIC), Salamanca, Spain

## Abstract

A growing need to evaluate risk-adapted treatments in multiple myeloma (MM) exists. Several clinical and molecular scores have been developed in the last decades, which individually explain some of the variability in the heterogeneous clinical behavior of this neoplasm. Recently, we presented Iacobus-50 (IAC-50), which is a machine learning-based survival model based on clinical, biochemical, and genomic data capable of risk-stratifying newly diagnosed MM patients and predicting the optimal upfront treatment scheme. In the present study, we evaluated the prognostic value of the IAC-50 gene expression signature in an external cohort composed of patients from the Total Therapy trials 3, 4, and 5. The prognostic value of IAC-50 was validated, and additionally we observed a better performance in terms of progression-free survival and overall survival prediction compared with the UAMS70 gene expression signature. The combination of the IAC-50 gene expression signature with traditional prognostic variables (International Staging System [ISS] score, baseline B2-microglobulin, and age) improved the performance well above the predictability of the ISS score. IAC-50 emerges as a powerful risk stratification model which might be considered for risk stratification in newly diagnosed myeloma patients, in the context of clinical trials but also in real life.

## INTRODUCTION

As the treatment landscape of multiple myeloma (MM) evolves, a growing interest exists to develop risk-adapted therapeutic schemes based on personalized drug combinations and variable treatment durations.^1^ Most used risk scores, such as the International Staging System (ISS) and the Revised ISS (R-ISS),^2,3^ though extensively validated, have limited accuracy in survival prediction, and assign a large fraction of the patients to an intermediate risk group whose outcomes are largely uncertain. On the other side, and even in properly stratified patients, optimal drug combination schemes and duration of therapies are unknown. All these factors contribute to the fact that, unfortunately, some patients will fail to respond or relapse earlier than expected after standard upfront drug combination schemes, which precludes a reduced survival.^4^

Therefore, a growing number of trials are evaluating innovative strategies based on risk and response-adapted therapies. One such strategy relies on the application of highly effective treatments (4 and even 5 drug combinations including monoclonal antibodies) to patient populations with high-risk factors, such as an elevated number of circulating plasma cells in peripheral blood or high-risk cytogenetics.^5,6^ Another strategy, explored in the MASTER trial, evaluates the possibility of adapting the intensity and duration of treatment to the sustained negative minimal residual disease (MRD).^7^

Different prognostic gene expression signatures have been developed in the field of MM.^8^ These scores have been designed with the intention to identify a group of high-risk patients. However, a great heterogeneity exists between the different signatures, and therefore a variable proportion of patients are assigned to the high-risk group. These models result of little utility to optimize treatment selection, and their application has been eminently restricted to the context of clinical trials. Recently, we presented the Iacobus-50 (IAC-50) prognostic and predictive model for newly diagnosed MM.^9^ This algorithm is a machine learning model of survival based on the integration of a 46-gene expression signature, B2-microglobulin, ISS stage, and first-line treatment scheme. IAC-50 proved to be highly discriminative to predict MM survival, and additionally, it was capable of predicting which of the different upfront treatment combinations maximized the probability of long-term survival for each patient.

The aim of this study was to validate the prognostic role of IAC-50 in an external cohort, and to compare its performance with the 70 gene expression signature developed by the University of Arkansas for Medical Sciences (UAMS).^10^ Our results indicate that the IAC-50 gene expression signature is reproducible and provides even more precise predictions about progression-free survival (PFS) and overall survival (OS) than the UAMS70 signature. Based on the results obtained, we discuss the future possibilities to introduce this model in the context of clinical trials and in clinical practice.

## MATERIALS AND METHODS

### Data origin and preprocessing

Patient data were produced by the UAMS team. Gene expression data were derived from CD138+ bone marrow cells of patients with newly diagnosed MM recruited in the total therapy (TT) trials 3, 4, and 5. Normalized gene expression estimates were downloaded from the Gene Expression Omnibus (GEO) database, with identification GSE136400.

TT3 applied 2 cycles of VTD-PACE (bortezomib, thalidomide, dexamethasone; 4-day continuous infusions of cisplatin, doxorubicin, cyclophosphamide, etoposide) as induction full doses and, at reduced doses, as consolidation after melphalan-based tandem transplantation.^11^ In 2008, UAMS70-defined low- and high-risk patients were assigned to TT4^12^ and TT5,^13^ respectively. TT4 was a phase 3 trial designed to determine whether low (L)-TT4 was associated with lesser toxicity by reducing induction and consolidation cycles after tandem transplants from 2 cycles each in standard (S)-TT4 to 1 cycle in L-TT4. To compensate for potentially reduced efficacy by this strategy, the transplant regimen was altered from a single melphalan dose of 200 mg/m^2^ (MEL200) to a fractionated 50 mg/m^2^/d × 4 days (MEL50x4) schedule with the addition of bortezomib and thalidomide (VTD) to exploit synergism between MEL50 × 4 and VTD. TT5 included 1 cycle of M-VTD-PACE induction with hematopoietic stem cell collection. This was followed by tandem autologous stem cell transplants with hybrid regimens Mel80 plus bortezomib, lenalidomide plus dexamethasone (VRD)-PACE. Sandwiched in between, 2 intertransplant cycles of MEL20-VTD-PACE were administered. Maintenance consisted of 3 years of alternating VRD and VMD (M, melphalan). As relapses were observed during maintenance, bortezomib was increased from 1.3 to 1.5 mg/m^2^ weekly.

Our purpose was to validate the prognostic capacity of the IAC-50 and to evaluate its performance in comparison with UAMS70. IAC-50 was designed with data from the CoMMpass database, and it also included the different groups of upfront treatment schemes employed. IAC-50 was able to identify the best upfront therapy for each patient as these were significantly associated with survival.^14^ However, in the present case, all trials tested similar combinations with little modifications, all of which were based on bortezomib, immunomodulatory drugs (IMIDs) and dexamethasone, so we could not evaluate the predictive value of IAC-50. Therefore, we focused on evaluating the prognostic value of the clinical and genomic parameters of IAC-50. Three hundred ninety-four patients had full annotation for all the clinical and biochemical variables included in IAC-50: age at diagnosis, b2-microglobulin, and ISS stage. Gene expression data were obtained using Affymetrix Human Genome U133 Plus 2.0 Array gene expression chips. Expression data for 39 of 46 of the genes included in the IAC-50 gene expression signature was available (Suppl. Table S1). Data from the remaining genes were not included in the gene expression chips.

### Statistical analysis

A random forest of survival model was created with the gene expression signature in the entire cohort.^15^ Harrel’s c-index was used to evaluate the discriminative power of the signature. Out-of-bag predictions derived from random forests were used to fit cox survival models. In these models, we computed time-dependent areas under the curve (AUCs) for OS and PFS prediction at different time points, with and without clinical and biochemical covariates.^16^ For comparison, we also tested the predictive power of UAMS70 with and without clinical and biochemical covariates in the same bootstrapped samples. Internal validation was performed using a bootstrapping without replacement algorithm (a cross-validation technique) with 500 cycles. In each cycle, 75% of the cohort was used for training and the remaining 25% of samples were used as a test set. Bootstrapped c-indexes were computed with the *pec* package using the bootstrapping cross-validation method with 500 cycles.^17^

## RESULTS

Baseline characteristics of the patients can be consulted in Table 1.

**Table 1.** Patients’ Baseline Characteristics

Variable	Results
ISS stages I/ II/ III	39.84%/31.82%/28.32%
Median B2-microglobulin	3.7 mg/dL
Median age at diagosis	59.9 y [32.4–75.2]
TT3 trial	59.65%
TT4 trial	37.59%
TT5 trial	3.26%
Median follow-up	91.1 mo

ISS = International Staging System; TT = total therapy.

### Prognostic impact of the IAC-50 gene expression signature and comparison with UAMS70 high-risk signature

We evaluated the model discriminative power of IAC-50 and UAMS70 using c-indexes. The IAC-50 gene expression signature achieved a bootstrapped c-index of 0.581 and 0.541 for OS and PFS prediction in the entire cohort, respectively. These results were superior to those of the UAMS70 high-risk gene expression signature (Table 2).

**Table 2.** C-indexes for OS and PFS Prediction in the Whole Cohort

	OS	PFS
Signature	Bootstrapped c-index	Bootstrapped c-index
IAC-50 GEP	0.581	0.541
UAMS70	0.569	0.534

IAC-50 = Iacobus-50; OS = overall survival; PFS = progression-free survival.

**Figure 1.**
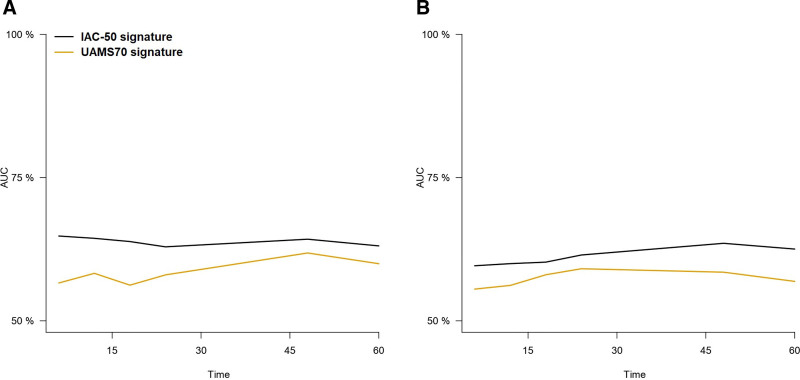
**Representation of time-dependent AUCs for the prediction of OS (A) and PFS (B) for the IAC-50 and UAMS70 gene expression signatures.** AUCs =areas under the curve; IAC-50 = Iacobus-50; OS = overall survival; PFS = progression-free survival.

We therefore sought to evaluate the performance of IAC-50 to predict PFS and OS at 6, 12, 18, 24, 48 and 60 months after diagnosis using time-dependent AUCs. Notably, the IAC-50 signature was superior to UAMS70 in the prediction of PFS and OS at most of the time points evaluated (Figure 1; Tables 3 and 4). Forty-eight-month PFS and OS predictions were similar overall between IAC-50 and the ISS score, but the UAMS70 high-risk profile showed a substantially lower prediction power. Notably, IAC-50 was superior to UAMS70 in the prediction of OS among patients <60, and it performed slightly better to UAMS70 in those ≥60 years (Suppl. Figure S1A and B, Suppl. Table S2). IAC-50 outperformed UAMS70 in the prediction of OS among ISS II and III patients, but not in the ISS I group (Suppl. Figure S2). In a similar fashion, we observed that IAC-50 was substantially better than UAMS70 in the prediction of PFS only among younger (<60 years) patients (Suppl. Figure S3C and D, Suppl. Table S3). The IAC-50 score also appeared to perform better than UAMS70 for PFS prediction in ISS I and III groups, but not in the ISS II group (Suppl. Figure S4).

**Table 3.** Cross-validated Time-dependent AUCs of the Different Models for the Prediction of OS at 6, 12, 18, 24, 48, and 60 Months

Model	6 mo	12 mo	18 mo	24 mo	48 mo	60 mo
ISS	0.713	0.709	0.676	0.655	0.658	0.676
IAC-50 GEP	0.648	0.644	0.638	0.629	0.642	0.631
UAMS70	0.566	0.583	0.562	0.580	0.619	0.600
IAC-50 GEP + ISS +B2-mg + Age	0.805	0.822	0.789	0.780	0.729	0.736
UAMS70 + ISS +B2-mg + Age	0.774	0.803	0.759	0.772	0.734	0.736

AUCs = areas under the curve; IAC-50 = Iacobus-50; ISS = International Staging System; OS = overall survival; PFS = progression-free survival.

**Table 4.** Cross-validated Time-dependent AUCs of the Different Models for the Prediction of PFS at 6, 12, 18, 24, 48, and 60 Months

Model	6 mo	12 mo	18 mo	24 mo	48 mo	60 mo
ISS	0.747	0.667	0.656	0.663	0.649	0.633
IAC-50 GEP	0.596	0.600	0.602	0.615	0.635	0.625
UAMS70	0.555	0.562	0.58	0.591	0.585	0.569
IAC-50 GEP + ISS +B2-mg + Age	0.782	0.704	0.694	0.703	0.702	0.664
UAMS70 + ISS +B2-mg + Age	0.791	0.714	0.714	0.719	0.706	0.670

AUCs = areas under the curve; IAC-50 = Iacobus-50; ISS = International Staging System; OS = overall survival; PFS = progression-free survival.

### Evaluation of IAC-50 gene expression signature with prognostic clinical and biochemical covariates

**Figure 2.**
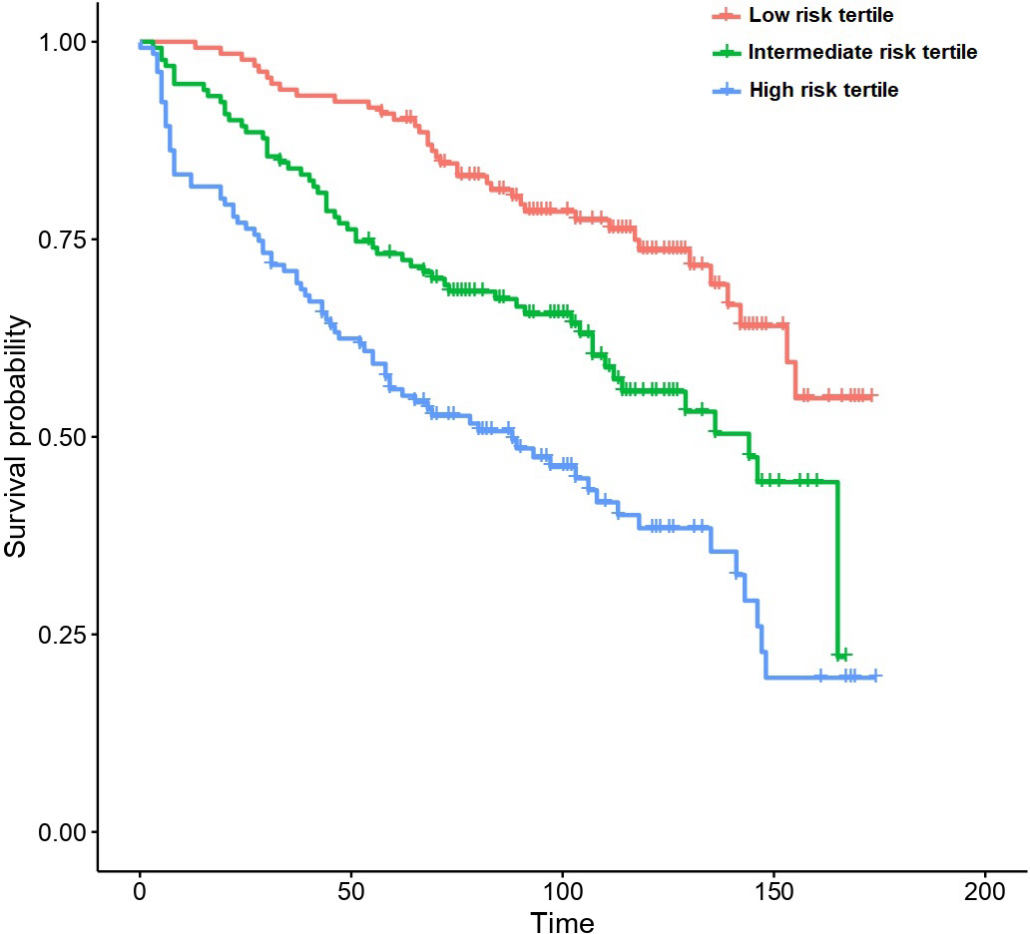
Patient survival according to the risk tertiles predicted by the IAC-50 signature plus ISS score, B2-microglobulin and age.

The integration of IAC-50 and UAMS70 with ISS, B2-microglobulin, and patient age largely improved the performance of any of these signatures in the prediction of PFS and OS (Figure 2; Tables 3 and 4). Importantly, these predictions were substantially better than those obtained from the ISS and the R-ISS score as an isolated prognostic model. Additionally, the R-ISS score appeared prognostically inferior to the ISS in this dataset (Suppl. Figure S5).

**Figure 3.**
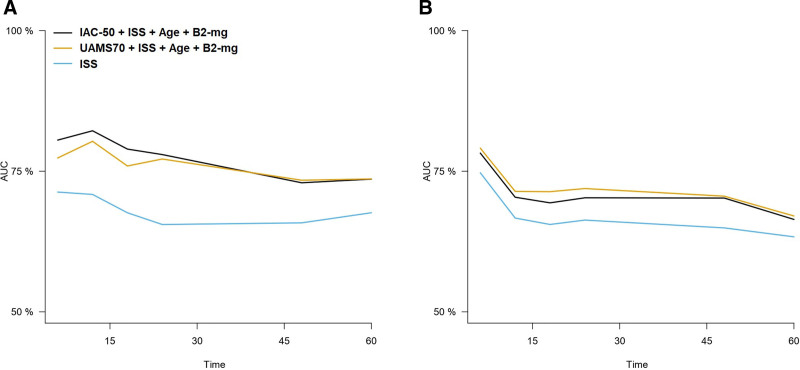
**Representation of time-dependent AUCs for the prediction of OS (A) and PFS (B) for the models considering IAC-50 and UAMS70 gene expression signatures plus ISS score, B2-microglobulin and age.** AUCs = areas under the curve; IAC-50 = Iacobus-50; ISS = International Staging System; OS = overall survival; PFS = progression-free survival.

The multivariate model including IAC-50 was superior to the model including UAMS70 for the prediction of overall survival, particularly in the first 2 years after diagnosis (Figure 3). This was particularly true for younger patients (<60 years), but no differences between both models were observed among older patients (Suppl. Figure S1C and D, Suppl. Table S2). Concerning PFS, we observed that, although in the global cohort there was a similar performance between both models (with a minor overperformance of UAMS70), there were again differences by age stratum: IAC-50 was superior among patients <60 years, whereas UAMS70 was superior in older individuals (Suppl. Figure S3C and D, Suppl. Table S3).

## DISCUSSION

The progressive untangling of cancer genomics coupled with advanced information technologies brings the opportunity to optimize risk stratification in MM with the possibility of implementing risk-adapted therapeutic strategies. The new risk stratification models that are emerging, such as IAC-50, need to validate their performance in additional external cohorts, ideally in comparison with other well-established risk stratification scores. In the present work, we have implemented IAC-50 in patients recruited in the TT trials 3–5, which included bortezomib and IMID-based triplets in newly diagnosed MM. Our results indicate not only that the IAC-50 gene expression signature is reproducible, but also it is superior to the UAMS70 high-risk signature in the prediction of both PFS and OS. Moreover, the combination of IAC-50 gene expression signature with traditional covariates originally included in the model (ISS, age, and B2-microglobulin) also outperforms UAMS70 in the prediction of OS, particularly in the first 2 years after diagnosis.

Prognostic gene expression profiles in MM have been developed with the aim of identifying subgroups of high-risk patients.^18^ These signatures are very heterogeneous in composition, and classify a remarkably different proportion of patients as high-risk. Additionally, as new and more effective drugs are being developed for the treatment of MM, the identification of low-risk patients is important to evaluate the possibility of achieving optimal disease responses with less intensive schemes than high-risk patients. Indeed, most trials evaluate the performance of treatment schemes by subgroups of risk, and even some relevant trials have been focused on standard or high-risk patients.^19–21^ However, the identification of such patients has not been the focus of traditional gene expression signatures. On the contrary, IAC-50 has been designed as a survival predictor, which is substantially more versatile because it can provide a precise and personalized risk prediction, enabling the identification of different strata of risk for patients with newly diagnosed MM at a glance. Our confrontation with the UAMS70 high-risk profile suggests that our approach yields superior prognostic results. Nonetheless, comparison with other signatures is needed, and particularly with SKY-92,^22^ as this has been observed to perform even better than UAMS70 by some authors.^23^

Notably, optimal risk stratification is obtained by combining molecular profiles with clinical risk scores. In this line, different studies have already evaluated the role of several gene expression signatures combined with the ISS and the R-ISS.^22,23^ However, compelling evidence indicates the limited performance of R-ISS over the ISS score, a fact which was also observed in this study.^24^ As a consequence, new prognostic scores are being proposed, such as the R2-ISS, the Mayo Additive Staging System (MASS) and unsupervised machine learning classifications proposed by the Spanish Myeloma Group (GEM/Pethema).^25–27^ These new scores propose the inclusion of other prognostic variables (eg, 1q amplification and Durie-Salmon staging) coupled with the definition of new patient groups based on advanced data analytics (additive scores and machine learning models). Such scores need to be evaluated in future studies and compared with each other. Furthermore, it will be important to incorporate performance status metrics in the models, as molecular profiles loose their importance in older unfit individuals with hematological cancer because of treatment toxicities.^28^ Indeed, this could be the reason behind the poor performance of both molecular prognostic signatures in older patients observed in this study. Importantly for the case, a comparison of the different molecular signatures with the same set of clinical predictor scores and considering frailty and/or comorbidity is needed, so as to derive the optimal combinations of variables. The adequate integration of clinical and molecular risk features will be of the utmost importance in order to produce a net gain of accuracy that can justify the implementation of molecular tests in clinical trials and in real life.

The present study has some limitations. First, only 39 of the 46 genes included in the IAC-50 signature were available for analysis. In our opinion, this should be viewed as a strength of the analysis because missing transcripts might even increase the precision of IAC-50. Second, the UAMS70 score was originally developed in patients from the TT 2 and 3 trials.^11^ On the contrary, IAC-50 was developed taking information from patients treated with a variety of upfront schemes from a different study. It is possible that UAMS70 predictions in this cohort are overoptimistic because of 2 factors: (1) some degree of overfitting to this particular cohort and (2) a skewed prediction accuracy towards patients treated with IMID plus dexamethasone or bortezomib, IMID plus dexamethasone-based upfront schemes. In both cases, the UAMS70 signature would be biased toward more positive results. Another relevant issue is the fact that clinical characteristics (and particularly age) explain a large part of mortality, overriding the role of biological variables when very heterogeneous cohorts are considered. Ideally, these scores should be evaluated in very homogeneous patient subgroups with similar age and comorbidities, probably in the context of well-designed clinical trials. This would provide a more sophisticated approximation about the role of biological risk in MM prognostication. Furthermore, the treatment schemes evaluated in these trials are not the standard of care in MM at the present moment, and therefore, evaluation of the molecular predictors in more recent populations should be pursued. Finally, we did not evaluate the predictability of the IAC-50 model, which was described in the original paper. This was because all treatments evaluated in the TT trials 3–5 were combinations of IMIDS, bortezomib, and dexamethasone, and indeed, we observed no association between overall survival and trial assignment.

In conclusion, we have externally validated the prognostic role of the IAC-50 gene expression signature in patients from the TT 3–5 trials. The accuracy of this signature was superior to UAMS70 in the prediction of overall survival and PFS. Combining the signature with traditional prognostic variables improved the performance well above the predictability of the ISS score. Future approaches should be pursued to compare this signature with additional key players in the field, such as SKY-92. IAC-50 should be considered for risk stratification in real life and, particularly, within the context of clinical trials.

## DISCLOSURES

MVMM has received honoraria for lectures and participation in advisory boards from Janssen, Celgene-BMS, Amgen, Takeda, Abbvie, GSK, Adaptive, Roche, Seatle Genetics, Pfizer, and Regeneron. AMO has received honoraria for lectures and participation in advisory boards from Janssen and AstraZeneca. AMO has received research grants from Roche and Celgene-BMS. MSGP has received honoraria for lectures and participation in advisory boards from Janssen, Amgen, Celgene-BMS, Takeda, Sanofi, and GSK. All the other authors have no conflicts of interest to disclose.

## AUTHOR CONTRIBUTIONS

AMO, JADA, and BAR developed the research. AMO, MSGP, and MVMM made suggestions and wrote the paper.

## ACKNOWLEDGMENTS

The authors wish to thank the Supercomputing Center of Galicia (CESGA) and the University of Arkansas for Medical Sciences for sharing the data, respectively.

## Supplementary Material


