# Supplementary material for: Prognostic Stratification of Multiple Myeloma Using Clinicogenomic Models: Validation and Performance Analysis of the IAC-50 Model
Source: Hemasphere. 2022 Aug 2;6(8):e760. doi: 10.1097/HS9.0000000000000760 (PMC9348861; doi:10.1097/HS9.0000000000000760)
Supplement: Supplementary file 1 [file hs9-6-e760-s001.pdf]

**Supplementary Table 1.** Genes included in the predictor.			
ACTL6B	*FGF1*	*MTMR9*	*TAF4B*
ARHGEF4	*FHOD1*	*PES1*	*TLL1*
CDCA5	*HPGD*	*PKMYT1*	*UBE2C*
CFAP45	*ITGA7*	*POLDIP3*	*UBE2S*
CLSTN3	*KIAA0101*	*PTPRR*	*UBE2V2*
DDX51	*LIMA1*	*RBP5*	*UNC13C*
DPF1	*MCM10*	*SHROOM3*	*WEE1*
ECHS1	*MCM4*	*SLC1A7*	*WNT9A*
ELOVL6	*MDM2*	*SNAP91*	*ZC3H18*
EXO1	*MRPL3*	*SRGN*	
