# Supplementary figures and images for: Prognostic Stratification of Multiple Myeloma Using Clinicogenomic Models: Validation and Performance Analysis of the IAC-50 Model
Source: Hemasphere. 2022 Aug 2;6(8):e760. doi: 10.1097/HS9.0000000000000760 (PMC9348861; doi:10.1097/HS9.0000000000000760)

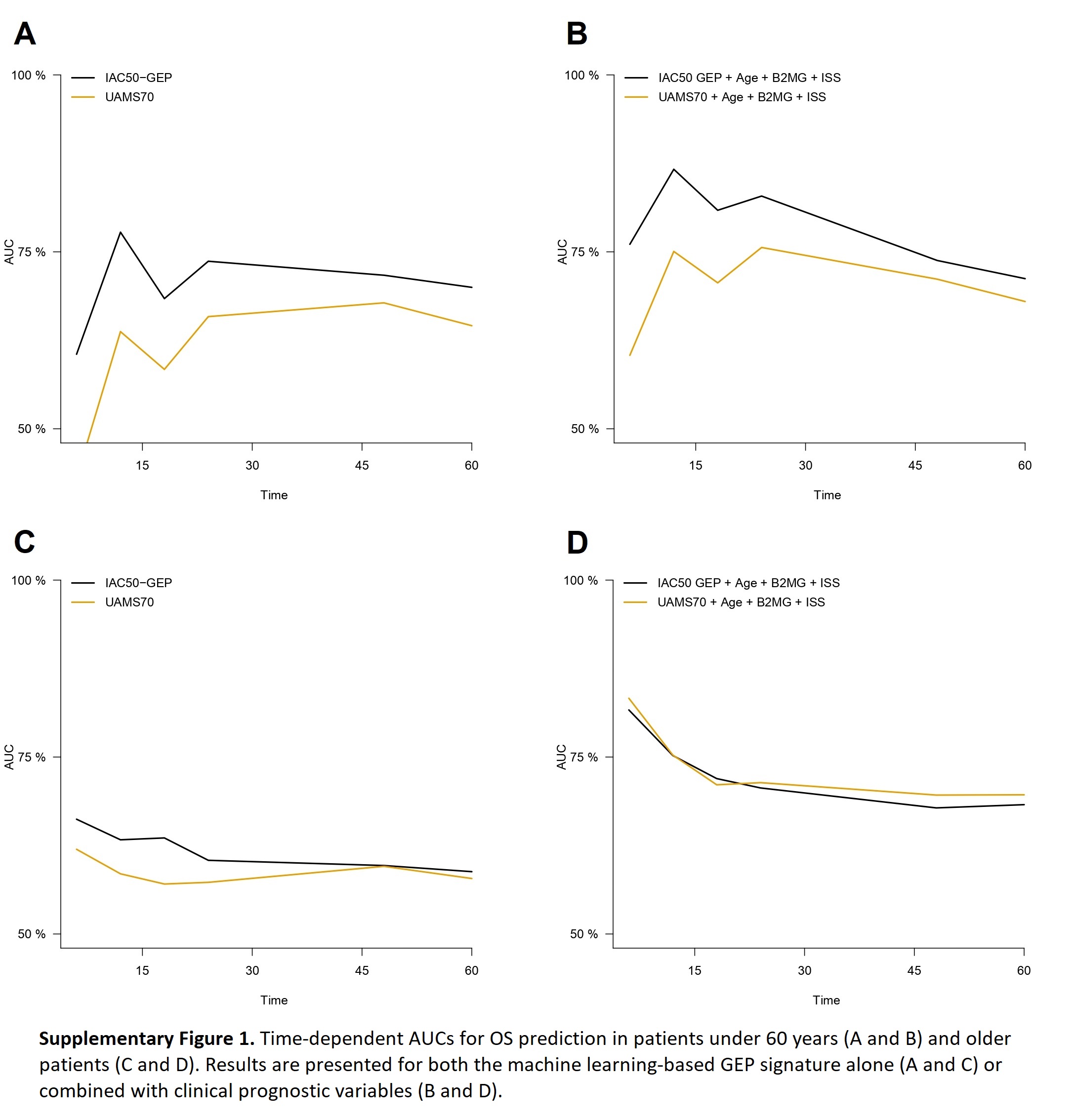

Supplement: Supplementary file 2 [file hs9-6-e760-s002.jpg]

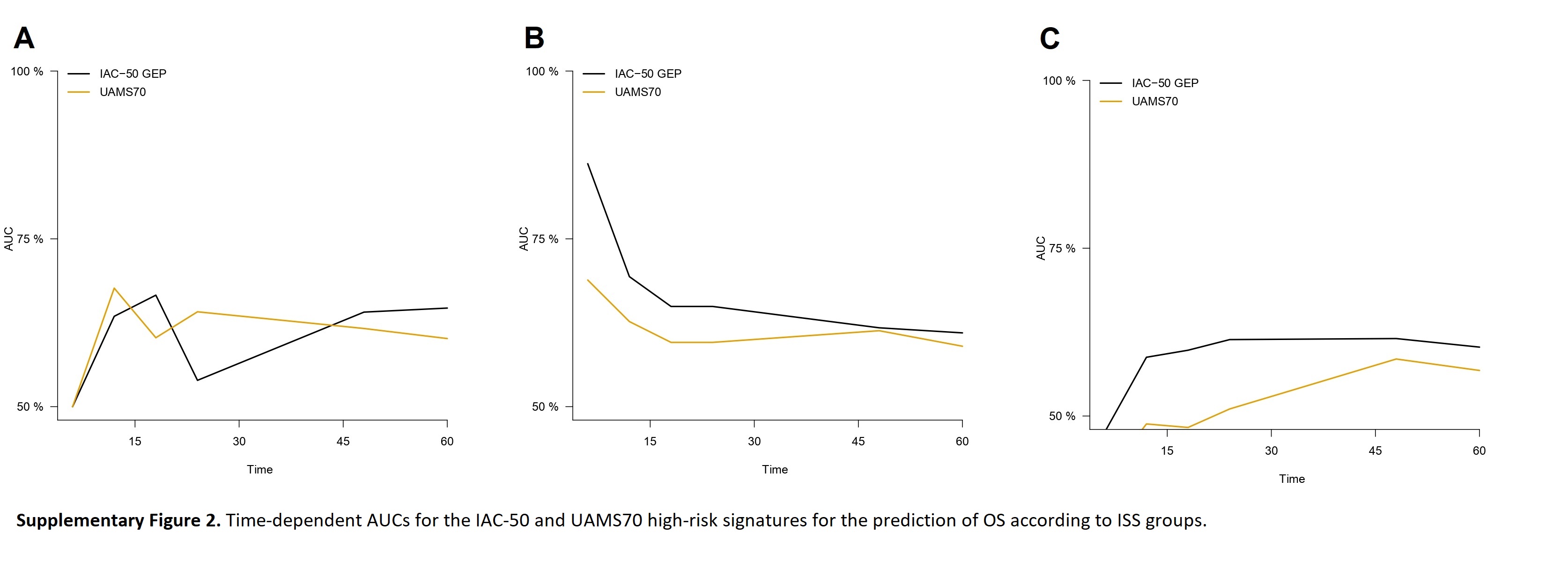

Supplement: Supplementary file 4 [file hs9-6-e760-s004.jpg]

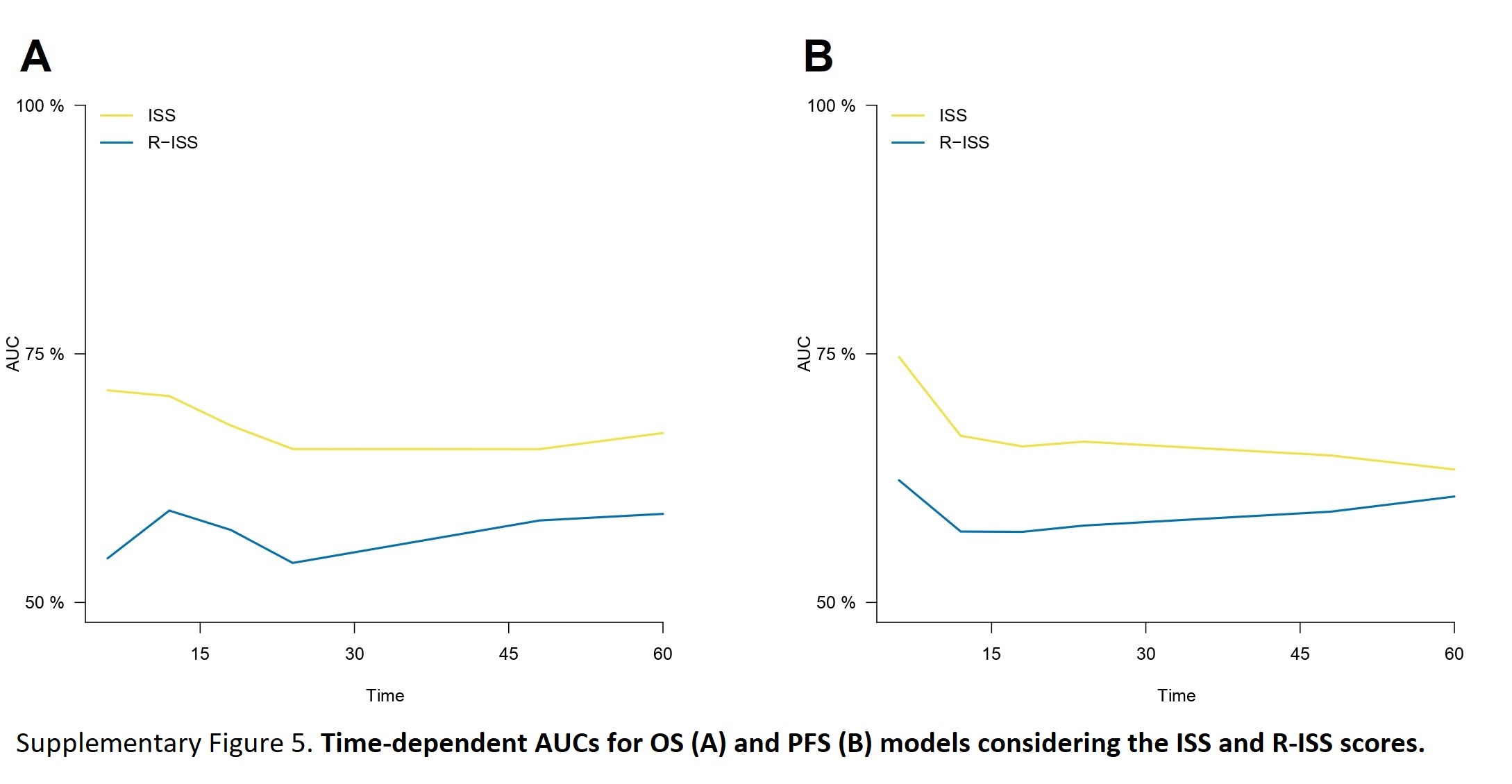

Supplement: Supplementary file 5 [file hs9-6-e760-s005.jpg]

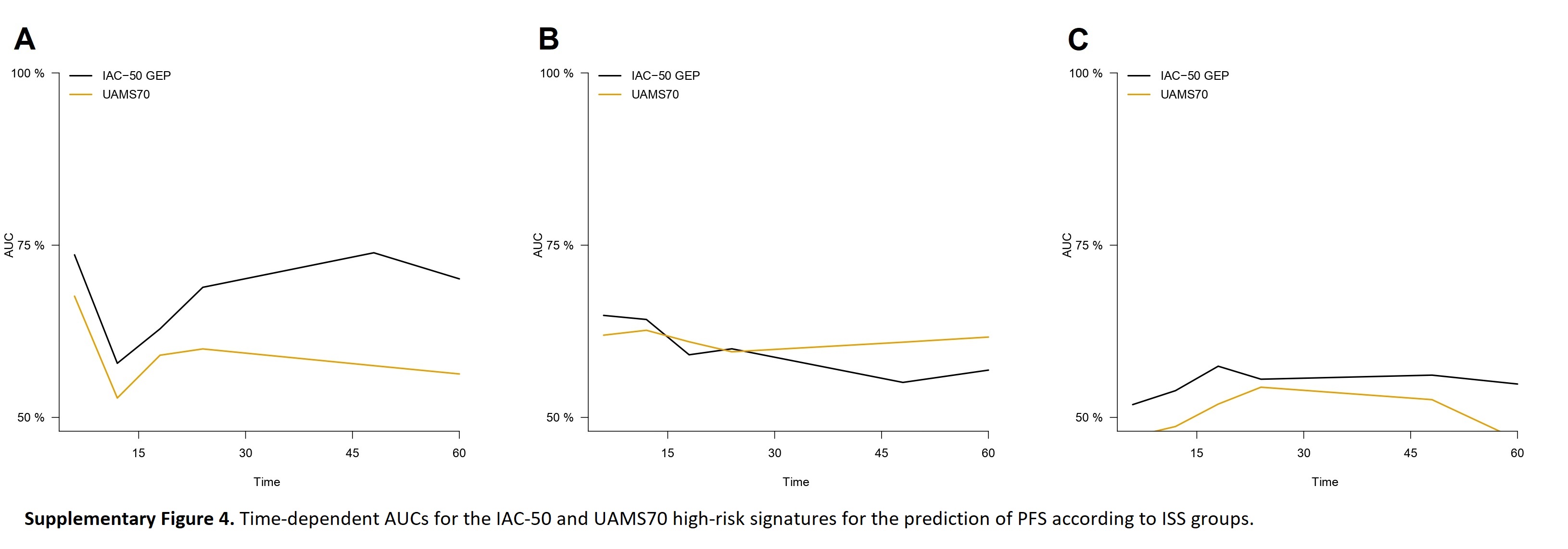

Supplement: Supplementary file 7 [file hs9-6-e760-s007.jpg]
