# Supplementary material for: Prognostic Stratification of Multiple Myeloma Using Clinicogenomic Models: Validation and Performance Analysis of the IAC-50 Model
Source: Hemasphere. 2022 Aug 2;6(8):e760. doi: 10.1097/HS9.0000000000000760 (PMC9348861; doi:10.1097/HS9.0000000000000760)
Supplement: Supplementary file 3 [file hs9-6-e760-s003.pdf]

Supplementary Table 2. Cross-validated time-dependent AUCs of the different models for the prediction of OS at 6, 12, 18, 24, 48 and 60 months by age strata.

>=60 years

Model	Time	AUC
IAC-50 GEP	6	0,662
IAC-50 GEP	12	0,633
IAC-50 GEP	18	0,636
IAC-50 GEP	24	0,604
IAC-50 GEP	48	0,597
IAC-50 GEP	60	0,588
UAMS70	6	0,62
UAMS70	12	0,585
UAMS70	18	0,571
UAMS70	24	0,573
UAMS70	48	0,596
UAMS70	60	0,578
IAC-50 GEP + ISS + B2-mg + Age	6	0,816
IAC-50 GEP + ISS + B2-mg + Age	12	0,752
IAC-50 GEP + ISS + B2-mg + Age	18	0,72
IAC-50 GEP + ISS + B2-mg + Age	24	0,706
IAC-50 GEP + ISS + B2-mg + Age	48	0,678
IAC-50 GEP + ISS + B2-mg + Age	60	0,683
UAMS70 + ISS +B2-mg + Age	6	0,833
UAMS70 + ISS +B2-mg + Age	12	0,753
UAMS70 + ISS +B2-mg + Age	18	0,711
UAMS70 + ISS +B2-mg + Age	24	0,714

< 60 years

Model	Time	AUC
IAC-50 GEP	6	0,605
IAC-50 GEP	12	0,778
IAC-50 GEP	18	0,684
IAC-50 GEP	24	0,737
IAC-50 GEP	48	0,717
IAC-50 GEP	60	0,7
UAMS70	6	0,43
UAMS70	12	0,637
UAMS70	18	0,584
UAMS70	24	0,658
UAMS70	48	0,678
UAMS70	60	0,646
IAC-50 GEP + ISS + B2-mg + Age	6	0,761
IAC-50 GEP + ISS + B2-mg + Age	12	0,867
IAC-50 GEP + ISS + B2-mg + Age	18	0,809
IAC-50 GEP + ISS + B2-mg + Age	24	0,829
IAC-50 GEP + ISS + B2-mg + Age	48	0,738
IAC-50 GEP + ISS + B2-mg + Age	60	0,712
UAMS70 + ISS +B2-mg + Age	6	0,604
UAMS70 + ISS +B2-mg + Age	12	0,751
UAMS70 + ISS +B2-mg + Age	18	0,706
UAMS70 + ISS +B2-mg + Age	24	0,756

UAMS70 + ISS +B2-mg + Age	48	0,696
UAMS70 + ISS +B2-mg + Age	60	0,697

UAMS70 + ISS +B2-mg + Age	48	0,711
UAMS70 + ISS +B2-mg + Age	60	0,68
