# Supplementary material for: Prognostic Stratification of Multiple Myeloma Using Clinicogenomic Models: Validation and Performance Analysis of the IAC-50 Model
Source: Hemasphere. 2022 Aug 2;6(8):e760. doi: 10.1097/HS9.0000000000000760 (PMC9348861; doi:10.1097/HS9.0000000000000760)
Supplement: Supplementary file 6 [file hs9-6-e760-s006.pdf]

Supplementary Table 3. Cross-validated time-dependent AUCs of the different models for the prediction of PFS at 6, 12, 18, 24, 48 and 60 months by age strata.

>= 60 years

Model	Time	AUC
IAC-50 GEP	6	0,626
IAC-50 GEP	12	0,56
IAC-50 GEP	18	0,567
IAC-50 GEP	24	0,603
IAC-50 GEP	48	0,6
IAC-50 GEP	60	0,622
UAMS70	6	0,586
UAMS70	12	0,555
UAMS70	18	0,582
UAMS70	24	0,608
UAMS70	48	0,578
UAMS70	60	0,559
IAC-50 GEP + ISS + B2-mg + Age	6	0,805
IAC-50 GEP + ISS + B2-mg + Age	12	0,687
IAC-50 GEP + ISS + B2-mg + Age	18	0,689
IAC-50 GEP + ISS + B2-mg + Age	24	0,704
IAC-50 GEP + ISS + B2-mg + Age	48	0,717
IAC-50 GEP + ISS + B2-mg + Age	60	0,722
UAMS70 + ISS +B2-mg + Age	6	0,82
UAMS70 + ISS +B2-mg + Age	12	0,708
UAMS70 + ISS +B2-mg + Age	18	0,726
UAMS70 + ISS +B2-mg + Age	24	0,738

< 60 years

Model	Time	AUC
IAC-50 GEP	6	0,574
IAC-50 GEP	12	0,736
IAC-50 GEP	18	0,713
IAC-50 GEP	24	0,682
IAC-50 GEP	48	0,689
IAC-50 GEP	60	0,652
UAMS70	6	0,499
UAMS70	12	0,623
UAMS70	18	0,613
UAMS70	24	0,598
UAMS70	48	0,613
UAMS70	60	0,592
IAC-50 GEP + ISS + B2-mg + Age	6	0,626
IAC-50 GEP + ISS + B2-mg + Age	12	0,732
IAC-50 GEP + ISS + B2-mg + Age	18	0,694
IAC-50 GEP + ISS + B2-mg + Age	24	0,677
IAC-50 GEP + ISS + B2-mg + Age	48	0,69
IAC-50 GEP + ISS + B2-mg + Age	60	0,622
UAMS70 + ISS +B2-mg + Age	6	0,597
UAMS70 + ISS +B2-mg + Age	12	0,647
UAMS70 + ISS +B2-mg + Age	18	0,618
UAMS70 + ISS +B2-mg + Age	24	0,611

UAMS70 + ISS +B2-mg + Age	48	0,739
UAMS70 + ISS +B2-mg + Age	60	0,728

UAMS70 + ISS +B2-mg + Age	48	0,642
UAMS70 + ISS +B2-mg + Age	60	0,575
